# Temporal Effects of High Fishmeal Diet on Gut Microbiota and Immune Response in *Clostridium perfringens*-Challenged Chickens

**DOI:** 10.3389/fmicb.2018.02754

**Published:** 2018-11-13

**Authors:** Ting Huang, Biao Gao, Wen-Lu Chen, Rong Xiang, Ming-Gui Yuan, Zhi-Hong Xu, Xin-Yu Peng

**Affiliations:** ^1^Institute of Animal Health, Guangdong Academy of Agricultural Sciences, Guangzhou, China; ^2^Key Laboratory of Livestock Disease Prevention of Guangdong Province, Guangzhou, China; ^3^Scientific Observing and Experimental Station of Veterinary Drugs and Diagnostic Techniques of Guangdong Province, Ministry of Agriculture, Guangzhou, China; ^4^Chinese Traditional Medicine Engineering Technology Research Center of Guangdong Province, Guangzhou, China

**Keywords:** *Clostridium perfringens*, high fishmeal diet, gut microbiota, immune response, chickens

## Abstract

Necrotic enteritis (NE) caused by *Clostridium perfringens* is responsible for huge financial losses in the poultry industry annually. A diet highly supplemented with fishmeal is one factor predisposing chickens to the development of clinical NE. However, the effects of fishmeal-rich diets on the gut microbiota and immune response in chickens with *C. perfringens* challenge over the long-term are not well-understood. Here, a chicken NE model was established in which chickens were fed high fishmeal diet and subsequently infected with *C. perfringens* (FM/CP). Two control groups of chickens, one that was not infected and had a high fishmeal feeding (FM) and another group only infected with *C. perfringens* with basic diets (CP), were used as comparators. We analyzed the gut microbiota and immune response of the three groups at the age of 20, 24 [1 day post-infection (dpi)] and 30 days (7 dpi) using 16S rDNA sequencing and real-time PCR, respectively. We found that the composition of the gut microbiota had significant shifted in both the CP and FM/CP groups, although the CP group did not have intestinal lesions. The structure of the gut microbiota in *C. perfringens*-challenged chickens, independent of a high fishmeal diet, had the tendency to return to their non-infection state if the chickens no longer received *C. perfringens* challenge. Gut microbiota variation with time in challenged chickens with high fishmeal diet feeding was superimposed upon that of non-infected chickens with high fishmeal feeding. For the immune response, the relative expression of IL-8 in the ileum was significantly higher in infected chickens independent of high fishmeal feeding than in non-infected chickens. However, the expression of alpha 1-acid glycoprotein (AGP) and serum amyloid A (SAA) genes in chicken liver were significantly increased in FM/CP compared to the other groups. In conclusion, high fishmeal feeding induced significant changes to the structure of chicken gut microbiota over time and such changes provided an opening for *C. perfringens* infection to progress to NE. The relative expression of AGP and SAA in liver tissue may be used as diagnostic biomarkers for poultry NE but such an indication requires further investigation.

## Introduction

Necrotic enteritis (NE) is intestinal disease of poultry caused by *Clostridium perfringens* (*C. perfringens*) common in domestic poultry-producing countries and poses a risk to animal health. This disease has been documented to cause a loss of more than US $2 billion per year in the international poultry industry, with an average loss of $0.05 per chicken (Kaldhusdal et al., 1999; Van der Sluis, 2000). Recently, the true cost of NE to the producer has been elevated to US $6 billion in 2015, with a cost of more than $0.062 per chicken (Wade Ben, 2015).

*Clostridium perfringens* infection alone is not enough to induce NE. Predisposing factors are necessary to produce special conditions to induce disease. Usually, the third to fourth week of a chicken’s life provide the most susceptible conditions for the development of NE. Using high-protein diets containing fishmeal are one predisposing factor that have been extensively studied in experimental NE models (Wu et al., 2010; Fernandes da Costa et al., 2013). Now we know that high fishmeal diets have an abundancy of available nutrients which can promote *C. perfringens* proliferation (Drew et al., 2004). It has been also reported that fishmeal feeding alone as well as the combination of fishmeal feeding and *C. perfringens* infection could induce a significant shift in chicken gut microbiota (Stanley et al., 2014; Wu et al., 2014). However, it is not clear how such a high-protein diet promotes NE.

The expression of several different genes has been found to be associated with *C. perfringens* infection. Two receptors of intestinal epithelial cells, nucleotide-binding oligomerization domain (NOD) and Toll-like receptor (TLR) 2, were shown to be activated by peptidoglycan (PGN) (Wu et al., 2007; Lin et al., 2011). Moreover, PGN is the main cell wall component of *C. perfringens* which may activate these two receptors. However, the recognition of *C. perfringens* and the following immune responses are not well-understood. In addition, acute phase protein (APP) expression could be affected by microbial infection. Five APP have been found to have different expression in diseased chickens, alpha 1-acid glycoprotein (AGP), serum amyloid A (SAA), PIT54, ovotrasferrin (OVT), and C-reactive protein (CRP) (O’Reilly and Eckersall, 2014). It has been reported that different bacterial or viral infections could elevate corresponding APPs levels. For example, OVT was overexpressed in chickens infected with *Escherichia coli* (Rath et al., 2009). Therefore, the APPs may provide a target for poultry infection disease diagnostics.

The aim of the present study was to compare changes to the microbiota in chickens exposed to high fishmeal diets as well as *C. perfringens* challenge over time. Combination of these two factors were used to gain a better understanding of how high fishmeal affects the microbiome and the immune response in chickens with *C. perfringens* infection.

## Materials and Methods

### Animal Trial Design and Sample Collection

**FIGURE 1:**
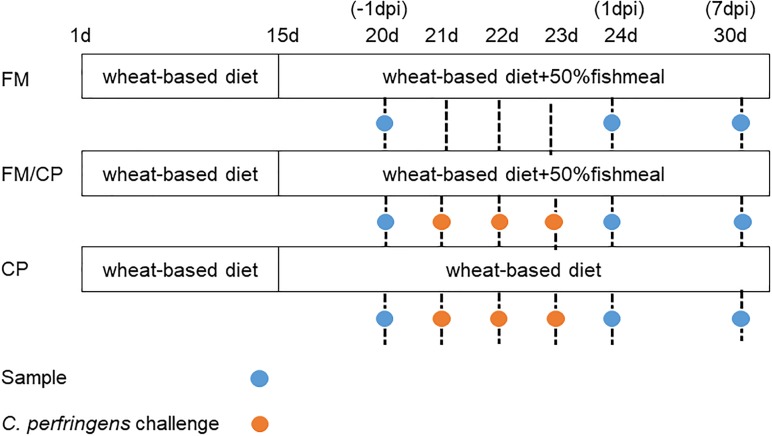
Animal experimental design. dpi, day post-infection.

One-day old Ross 308 commercial chickens (negative for *C. perfringens*) were purchased from a hatchery in Guangzhou City, Guangdong Province, China. They were kept in pens for 14 days and then were randomly assigned on the basis of weight to one of three experimental groups. Food and water were available *ad libitum* and the chickens were maintained with 12 h light/dark cycles. Animal trials were conducted at the poultry house located at the Institute of Animal Health, Guangdong Academy of Agricultural Sciences. Three groups (60 chickens per group) were designed: (1) a chicken NE model in which chickens were fed high fishmeal diet and subsequently infected with *C. perfringens* (FM/CP), (2) chickens without additional infection subject to a high fishmeal feeding (FM), and (3) chickens infected with *C. perfringens* under basic diet (CP). From day of age 1 to 14, all groups were fed an antibiotic-free chicken basic diet (Supplementary Table S1). From day 15 to 30, group FM and FM/CP were switched to a high-protein diet (Supplementary Table S1) and group CP was kept on the same diet as before. Group FM/CP and CP were orally gavaged in the corresponding diets twice per day with actively growing concentration of 7–9 × 10^8^ cfu/ml *C. perfringens* cpnetBF (Stanley et al., 2012) on days 21–23. The strain *C. perfringens* cpnetBF carries the *netb* gene isolated from intestines of broiler chickens with necrotic enteritis in Guangdong Province, China. The strain was stored at -80°C in fluid thioglycollate broth (FT, Beckson, Dickinson and Company) supplemented with 30% glycerol. None of the chickens died during the experiment. This animal experimental design is shown in Figure 1.

Seven chickens from each group were euthanized by cervical dislocation for sampling at day of age 20, 24, and 30. Jejunum–ileum content samples were collected for subsequent DNA extraction. Ileal and liver tissues were collected for subsequent RNA isolation. Ileal tissues were collected from approximately 3 cm distal to Meckel’s diverticulum. The collected tissue were immediately frozen in liquid nitrogen.

All the experimental procedures involving chickens were approved by the guidelines and regulations of Guangdong Academy of Agricultural Sciences [No. SYXK (yue)-20171211].

### Intestinal Lesion Score

The lesion scores of the small intestine (duodenum to ileum) were observed from 10 replications for three different groups as previous study (Keyburn et al., 2006): (0) no gross lesions; (1) thin or friable walls; (2) focal necrosis or ulceration (one to five foci); (3) focal necrosis or ulceration (6 to 15 foci); (4) focal necrosis or ulceration (16 or more foci); (5) patches of necrosis 2–3 cm long; (6) diffused necrosis typical of field cases.

### DNA Extraction and the Abundance of *C. perfringens* Detection

DNA from chickens’ jejunum–ileum content contents was extracted using QIAamp Fast DNA Stool Mini Kit (Qiagen, Valencia, CA, United States) according to the manufacturer’s instructions. Total DNA was quantified using a NanoDrop^®^ ND-2000 UV spectrophotometer (NanoDrop Technologies, Wilmington, DE, United States). The instrument measures absorbance at 260 nm (A260) to quantify DNA in samples, at 280 nm (A280) to verify protein contamination and at 230 nm (A230) for determining contamination by phenol. Only DNA samples with A260/A280 ratio as 1.7 and A260/A230 > 1.8 were used for further analysis (Li et al., 2014). The extracts were stored at -20°C until use (Singh et al., 2015).

Quantitative PCR reaction was performed with intestinal content DNA temple in triplicate using SYBR Premix Ex Taq (TAKARA Bio, Otsu, Japan). The *C. perfringens* genes values were normalize to the 16S rRNA gene. The relative abundance of *C. perfringens* in the intestine were calculated based on the value of the 16S rRNA gene using 2^-ΔCt^ method. The primers used for qPCR of *C. perfringens* and 16S rRNA are showed in Supplementary Table S2.

### 16S rRNA Gene Sequencing

The V3+V4 hypervariable regions of 16S rDNA were PCR amplified from microbial DNA harvested from seven replications for three different groups at days 20, 24, 30 (Abrahamsson et al., 2014; Jakobsson et al., 2014). The gene-specific sequences for the 16S V3 and V4 region were performed by using primers 341F 5′-CCTACGGGNGGCWGCAG-3′ and 805R 5′-GACTACHVGGGTATCTAATCC-3′ (Abrahamsson et al., 2014; Jakobsson et al., 2014). The PCR conditions were as follows: one pre-denaturation cycle at 94°C for 4 min, 25 cycles of denaturation at 94°C for 30 s, annealing at 55°C for 45 s, and elongation at 72°C for 30 s, and one post-elongation cycle at 72°C for 5 min. The PCR amplicons were separated on 0.8% agarose gels and then extracted using QIAEX II gel extraction kit (Qiagen) according to the handbook. Only PCR products without primer dimers and contaminant bands were used for sequencing. Amplicons were purified using AMPure X using the manufacturer’s instructions (Beckman Coulter, Mississauga, ON, Canada). Bar-coded V3 and V4 amplicons were sequenced using the 2 × 300 paired-end method by Illumina MiSeq with a seven-cycle index read. Sequences processing was performed using QIIME (version 1.6.0) to get clean data. Sequences with an average Phred (Q) score lower than 30, with ambiguous bases or homopolymer runs exceeding 6 bp, primer mismatches or sequence lengths shorter than 100 bp were removed. The consensus sequence was generated by FLASH (Fast length Adjustment of Short reads, v1.2.11) as following: only sequences with an overlap longer than 10 bp and without any mismatches were assembled according to their overlap sequences. Reads that could not be assembled were discarded. Barcode and sequencing primers were trimmed from the assembled sequence. The high quality paired-end reads were combined to tags based on overlap. The tags were clustered to operational taxonomic unit (OTU) by software USEARCH (v7.0.1090). OTU representative sequences were taxonomically classified using Ribosomal Database Project (RDP) Classifier v2.2 trained on the Greengenes database.

### RNA Isolation and RT-qPCR of the Immune Genes

Using the EZNA^®^ Total RNA Isolation Kit (Omega Bio-Tek), RNA was isolate from ileal and liver tissue according to the manufacturer’s protocol. RNA was eluted in DEPC-treated water and stored at -80°C. Total RNA was quantified using a NanoDrop^®^ ND-2000 UV spectrophotometer (NanoDrop Technologies, Wilmington, DE, United States).

Reverse transcription was performed with M-MLV Frist Strand cDNA Synthesis Kit (Omega Bio-Tek), following the manufacturer’s protocol. Quantitative PCR reaction was performed with cDNA temple in triplicate using SYBR Premix Ex Taq (TAKARA Bio, Otsu, Japan). The target genes values were normalize to the housekeeping gene encoding glyceraldehyde-3-phosphate dehydrogenase (GAPDH). The relative mRNA level of each target gene were calculated based on the expression of the GAPDH using 2^-ΔCt^ method. The primers used for qPCR of interleukin-8 (IL-8), tumor necrosis factor α (TNF-α), Toll-like receptor 2 (TLR2), nucleotide-binding oligomerization domain 1 (NOD1), alpha1-acid glycoprotein (AGP), serum amyloid A (SAA), C- reactive protein (CRP), PIT54, ovotransferrin (OVT) and glyceraldehyde-3-phosphate dehydrogenase (GAPDH) are showed in Supplementary Table S2.

### Statistical Analysis

The relative expression of mRNA, the relative abundance of *C. perfringens* and the alpha diversity indices of Shannon and abundance-based coverage (ACE) among the three groups were tested using single-factor analysis of molecular variance ANOVA with a *p*-value of < 0.05 considered to represent significance. Genus and species abundance were compared using Kruskal–Wallis test with Benjamini–Hochberg *p*-value correction.

## Results

### Intestinal Lesion Score

**FIGURE 2:**
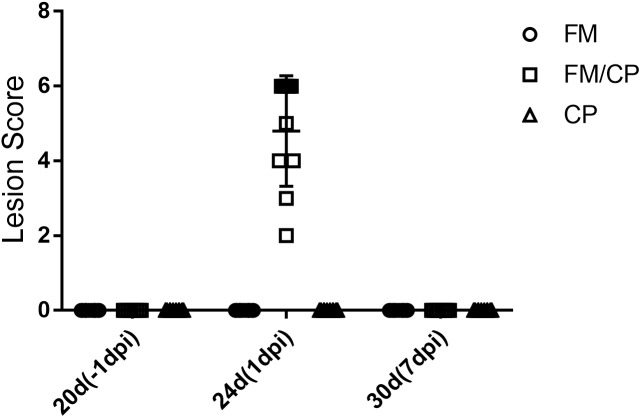
Intestinal lesion scores of chickens at each time point. dpi, day post-infection.

No intestinal lesions were observed in unchallenged chickens (Figure 2). In the challenged groups, only the group FM/CP had obvious lesions at 1 day post-infection (dpi). At 7 dpi, all the challenged chickens showed no intestinal lesions.

### *Clostridium perfringens* Colonization

**FIGURE 3:**
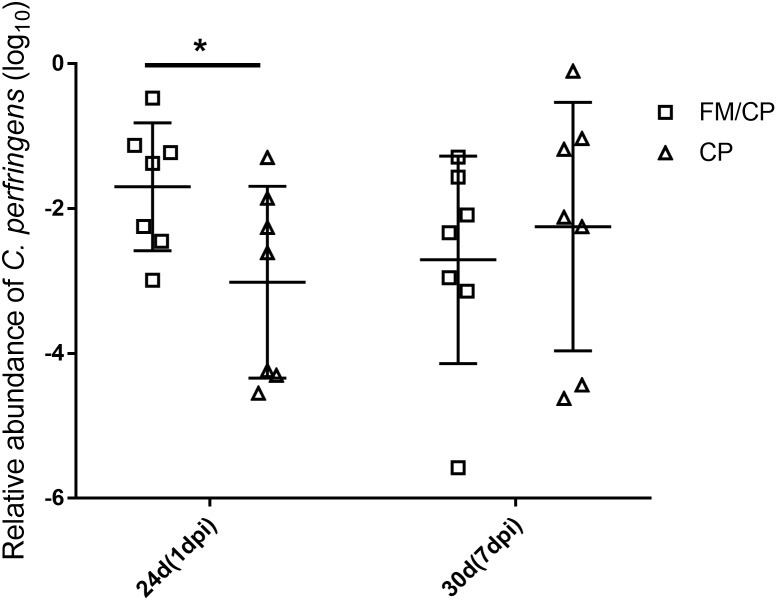
Viable relative abundance of *Clostridium perfringens* in the jejunum–ileum lumen. FM/CP and CP groups at day 20 and group FM remained negative for *C. perfringens* throughout the study. No significant differences between group FM/CP and CP at day 30 were observed (^∗^*p* > 0.05; ANOVA).

All chickens were negative for C. *perfringens* until experimental challenging and group FM remained detection negative for C. *perfringens* throughout the study (Figure 3). The relative abundance of *C. perfringens* in chickens with FM/CP was significantly higher than group CP at 1 dpi (*p* < 0.05). However, at 7 dpi, there was no significant difference between group FM/CP and CP.

### Immune Related Genes Expression

**FIGURE 4:**
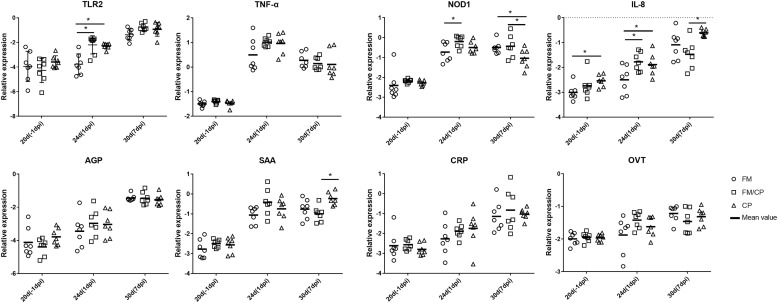
Relative change in expression of immune genes in ileum tissue. Relative gene expression represented as log_10_. Significant difference among three groups are indicated by an asterisk (ANOVA, ^∗^*p* < 0.05) for the expression of each genes at the corresponding time point. The Ct values of PIT54 in three groups was below the detection level. IL, interleukin; TNF-α, tumor necrosis factor α; TLR, toll-like receptor; NOD, nucleotide-binding oligomerization domain; AGP, alpha 1- acid glycoprotein; SAA, serum amyloid A; CRP, C- reactive protein; OVT, ovotransferrin; dpi, day post-infection.

**FIGURE 5:**
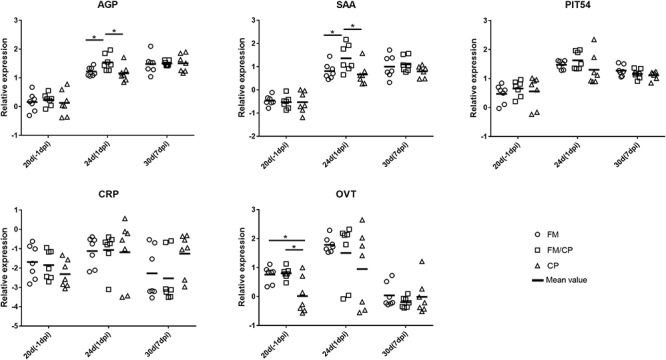
Relative change in expression of immune genes in liver tissue. Relative gene expression represented as log_10_. Significant differences among the three groups are indicated by an asterisk (ANOVA, ^∗^*p* < 0.05) for the expression of each gene at the corresponding time point. AGP, alpha 1- acid glycoprotein; SAA, serum amyloid A; CRP, C- reactive protein; OVT, ovotransferrin; dpi, day post-infection.

In order to investigate the effect of high fishmeal diet on the intestine and liver immune function in *C. perfringens* challenged chickens, the relative expression genes of cytokines, chemokine, Toll-like and NOD receptor and APPs was evaluated using the qPCR method (Figures 4, 5). At -1 dpi, the relative expression of all the detected immune genes had no significant difference among the three groups in ileum and liver tissue except the relative expression of OVT in liver and IL-8 in ileum. Moreover, the relative expression of TNF-α, AGP, CRP, and OVT in ileum tissue as well as PIT54 and CRP in liver tissue were not significantly different among the three groups at each time point. At 1 dpi, the relative expression of IL-8 in the ileum was significantly higher in infected chickens independent of high fishmeal diet as compared to non-infected chickens (*p* < 0.05). In addition, the relative expression of TNF-α, NOD1, AGP, SAA, CRP, and OVT had similar results as IL-8 expression among the three groups in ileum at same time points, without significant difference (*p* > 0.05). For liver tissue, the relative expression of AGP and SAA genes were significantly higher in group FM/CP compared to those in group FM and CP at 1 dpi. At 7 dpi, no significant changes were observed among three groups in liver and ileum tissue except IL-8, NOD1, and SAA in the ileum.

### Microbiota Composition

**FIGURE 6:**
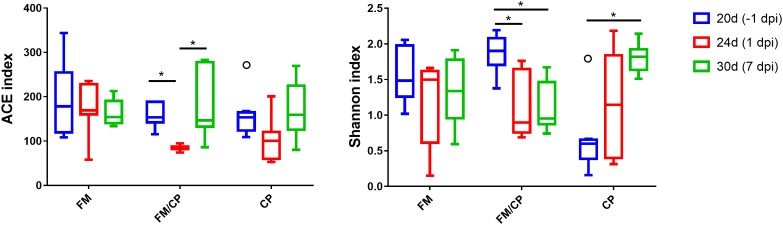
Estimates of alpha diversity for the gut microbiota from intestine content. ^∗^*p* < 0.05, ANOVA test.

Shannon and ACE indices were used to measure the alpha diversity (evenness and richness) of the gut microbiota. The FM group did not have significant changes in alpha diversity of microbiota in chickens from day of age 20 to 30 (*p* > 0.05, Figure 6). After challenging, the ACE indices in the group FM/CP were significantly lower at 1 dpi compared to that at -1 and 7 dpi. The FM/CP group had significantly lower Shannon indices at the challenge stage than at the stage baseline while the group CP showed the opposite.

**FIGURE 7:**
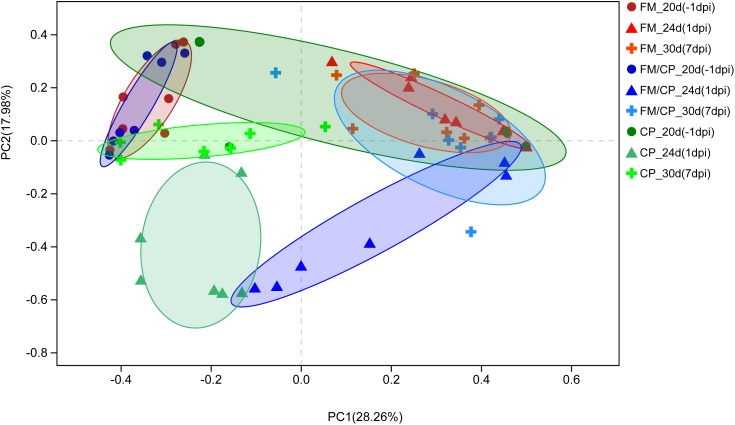
Principal coordinate analysis (PCoA) plot of Bray–Curtis indices for the gut microbiota of three different groups. The different degree red, blue, and green indicated group FM, FM/CP, and CP respectively. Circle: day 20 (–1 dpi); Triangle: day 24 (1 dpi); Plus sign: day 30 (7 dpi).

Principal coordinate analysis (PCoA) of Bray–Curtis indices demonstrated the difference in bacteria composition among the three experimental groups at age days 20, 24, and 30 (-1, 1, and 7 dpi, Figure 7). Before challenging, the structure of gut microbiota in group FM and FM/CP at -1 dpi was significantly different from stage-matched chickens in group CP. After challenging, the gut bacteria composition of group FM/CP and CP at 1 and 7 dpi were significantly different from corresponding group at -1 dpi. At 1 dpi, the PCoA plot showed clustering the gut bacteria with respect to three different groups. At 7 dpi, the gut bacterial composition of chickens in the group FM/CP and CP both had the tendency to return to an unchallenged stage.

**FIGURE 8:**
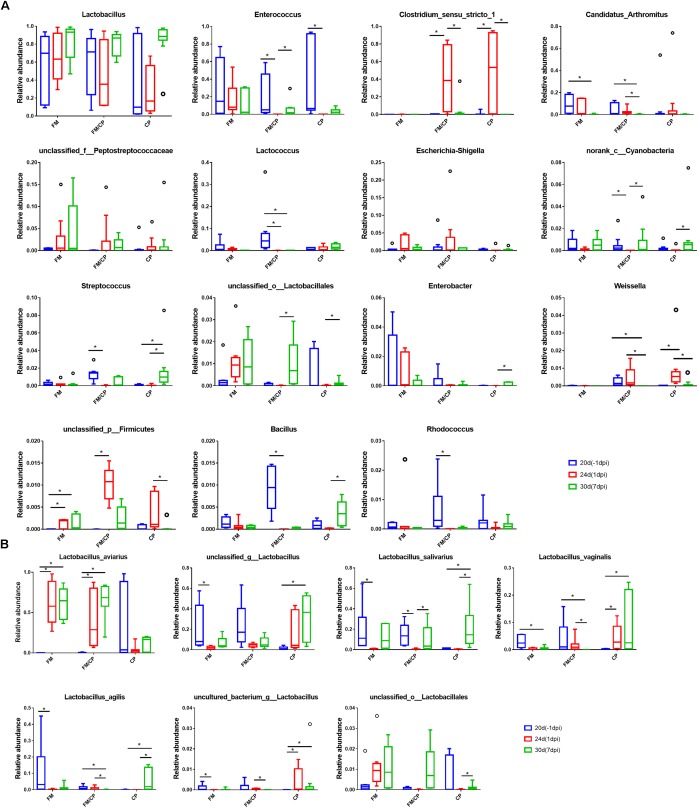
Comparison of bacterial composition using relative abundance. At the genus **(A)** and species **(B)** level. ^∗^*p* < 0.05, Kruskal–Wallis test.

The results of comparison of microbiota in each group showed that the relative abundance of genus *Candidatus Arthromitus* in the group FM and FM/CP was significantly lower at day 30 than that at day 20 (*p* < 0.05, Figure 8A), but group CP had no significant change. The unclassified_p_Firmicutes bacterial strain found in group FM/CP and FM was significantly higher at day 24 than that at day 20, moreover, this genus in the group FM/CP was significantly higher compared to that in the group FM at day 24. The relative abundance of *Enterococcus* in the group FM/CP and CP at 1 dpi was significantly lower than that at -1 dpi whereas the genus *Clostridium* sensu stricto 1 showed the opposite result (*p* < 0.05).

Although the total of genus *Lactobacillus* was predominant in the chickens gut bacterial composition were not significantly shifted from day 20 to day 30 in group FM, FM/CP and CP respectively (*p* > 0.05, Figure 8A), some species of *Lactobacillus* had significant changes (Figure 8B). The relative abundance of *Lactobacillus aviarius* (*L. aviarius*) in group FM and FM/CP at days 24 and 30 were significantly higher compared to day 20 (*p* < 0.05). Additionally, *L. aviarius* become the dominant species of *Lactobacillus* in group FM and FM/CP at day 24. All raw sequence were submitted to the Sequence Read Archive database at NCBI (Accession No. SRP159590).

## Discussion

It has been previously shown that *C. perfringens* challenge can shift the structure of gut microbiota in chickens (Stanley et al., 2012). However, the effect of fishmeal-rich diets on gut bacterial composition and the immune response during the development of NE have not been thoroughly investigated. To determine these effects, we studied the community structures and the relative expression of immune related genes in NE models with high fishmeal diet.

It was previously reported that long-term feeding of a high-protein diet could decrease the expression of TNF-α (Kitabchi et al., 2013) and feeding with a high fishmeal could increase the AGP concentration in the plasma of chickens (Takahashi et al., 2007). However, in this study, at day of age 20, we observed that the high fishmeal did not affect the expression of TNF-α in ileum or AGP in liver of non-infected chickens, but a high fishmeal diet did reduce the expression of IL-8 in ileum and increase the expression of OVT in the liver.

Remarkably, the recognition of *C. perfringens* by NOD and TLR2 receptor has been shown to lead to activation of downstream signaling pathways to induce chemokine (e.g., IL-8) and cytokine (e.g., TNF-α) expression (Girardin et al., 2003; Lu et al., 2009). *Eimeria maxima*/*C. perfringens* co-infection and *C. perfringens* infection alone with high fishmeal diet could up-regulated the IL-8 and TNF-α transcript level in chickens respectively (Lee et al., 2013; Li et al., 2018). Moreover, *E. maxima*/*C. perfringens* challenge significantly increased the expression of NOD1 gene in the intestine (Zhang et al., 2017). However, the TNF-α expression in chicken intestinal epithelial cells was not significantly affected by CP *in vitro* (Guo et al., 2015). In this study, we observed that *C. perfringens* infection independent of high fishmeal diet significantly increased the expression of TLR2, NOD1 and IL-8 in intestine tissue at 1 dpi, suggesting that *C. perfringens* infection independent of high fishmeal diet induced the expression of the above genes to increase.

Relative APP expression was higher in the liver compared with ileum, consistent with the previous report that the expression of APP genes was higher in liver than other tissue (Marques et al., 2017). AGP is a highly glycosylated protein secreted by hepatocytes (Murata et al., 2004). It plays important role in the early stage of inflammation and infection in chickens. For example, *Escherichia coli* as well as *Salmonella typhimurium* could significantly increase AGP expression (Takahashi et al., 1998; Adler et al., 2001). Moreover, Chamanza et al. (1999) demonstrated that perhaps SAA is a reliable APP for diagnosing inflammatory lesions in chickens. The expression of SAA also could be increased by bacterial and viral infection such as with *Pasteurella multocida*, *Staphylococcus aureus* and infectious bronchitis virus (Chamanza et al., 1999; Kovacs et al., 2007; Asasi et al., 2013). In our study, *C. perfringens* challenge with a high fishmeal diet, rather than infection alone, resulted in a higher intestinal lesion score and increased the relative abundance of *C. perfringens* at 1 dpi that was accompanied by an increase in AGP and SAA increase in liver. This suggested that the effect on the level of the AGP and SAA expression in response to the *C. perfringens* infection was dependent on a high fishmeal diet.

With regard to gut bacterial structure, the PCoA plot of Bray–Curtis indices demonstrated that chickens with high fishmeal diets were clustered and separated from the basic diet feeding chickens at day 20. This was consistent with other studies that showed high fishmeal diets had significant effects on the structure of the chickens gut microbiota (Stanley et al., 2014; Wu et al., 2014). Notably, with prolonged high fishmeal diet, PCoA showed a partition of chickens independent of *C. perfringens* challenge between early (20 days) and later (24 and 30 days) phases of the experiment. We hypothesized that the structure of the chicken gut microbiota undergoes transition with exposure to the high fishmeal over time and less shifting occurs after reaching a certain composition. Studies about the relationship between exposure time and fishmeal diet and the gut microbiota in chickens are rare and thus require further investigation.

In addition, *C. perfringens* challenge destabilized chickens gut microbiota independent of high fishmeal diet, and the structure of chicken gut bacteria had the tendency to return to the non-infection state when the chickens did not keep *C. perfringens* challenge. Our results were different from a study showed the stability of chickens intestinal microbiota is not influenced by the *C. perfringens* challenge alone (Stanley et al., 2014). Furthermore, in this study, the gut bacterial structure of group FM/CP were different from that of group CP at 1 dpi, which is corresponding to our results showed that *C. perfringens*-challenged chickens with high fishmeal feeding demonstrated significantly higher *C. perfringens* colonization level and higher lesion score compared to challenged alone chickens.

For gut bacterial composition, *Candidatus Arthromitus* (*C. Arthromitus*) are commensal bacteria in the family *Lachnospiraceae* which belong to the segmented filamentous bacteria (SFB) group (Thompson et al., 2012b). These bacteria can attach to the intestinal epithelium and modulated the host immune system (Thompson et al., 2012a). We observed that the high fishmeal could decrease abundance of *C. Arthromitus* however *C. perfringens* challenge alone did not affect the abundance of these organisms. A decreased fraction of *C. Arthromitus* was detected in the gut of broiler chickens with the combination *E. maxima* and *C. perfringens* or *E. maxima* alone infection (Stanley et al., 2014; Kim et al., 2015). Previous studies also reported that the genus *Lactobacillus* was predominant in chicken gut microbiota and the total genus *Lactobacillus* was not perturbed in chickens following high fishmeal diet feeding or *C. perfringens* challenge as also shown in the present study (Stanley et al., 2014; Yan et al., 2017). *L. aviarius* was one of the predominant *Lactobacillus* species in chickens found by Wang et al. (2014). Similarly, we found that the increase of *L. aviarius* in high fishmeal feeding chickens independent of *C. perfringens* challenge in this study was the result of high fishmeal diet and not *C. perfringens* challenge.

## Conclusion

Although *C. perfringens* challenge alone did not cause intestine lesions, it shifted the structure of the chicken gut microbiota. After infection, both *C. perfringens* challenge with a high fishmeal diet and *C. perfringens* challenge alone had the tendency to return to the non-infection stage when the chickens did not have persistent *C. perfringens* challenge. The temporal shift of gut microbiota structure in *C. perfringens*-challenged chickens with high fishmeal diet were superimposed upon the chickens with high fishmeal diet alone. Finally, we found that the expression of AGP and SAA in chicken liver tissue may be able to provide an assessment of poultry NE but such an application requires further investigation.

## Author Contributions

X-YP and Z-HX conceived of this study and participated in its design and coordination. TH designed the experiment, drafted the manuscript, and participated in the statistical analyses. TH, BG, W-LC, M-GY, and RX carried out animal experiments. TH and BG carried out the amplicon sequencing and qPCR. All authors read and approved the final manuscript.

## Conflict of Interest Statement

The authors declare that the research was conducted in the absence of any commercial or financial relationships that could be construed as a potential conflict of interest.
