# Supplementary material for: Temporal Effects of High Fishmeal Diet on Gut Microbiota and Immune Response in Clostridium perfringens-Challenged Chickens
Source: Front Microbiol. 2018 Nov 13;9:2754. doi: 10.3389/fmicb.2018.02754 (PMC6243065; doi:10.3389/fmicb.2018.02754)
Supplement: Supplementary file 1 [file Table_1.DOCX]

Table S1. Composition and nutritive levels of the diets (air-dry basis, %)

| Ingredients | Basic diets | High fishmeal diets |
| --- | --- | --- |
| Soya bean meal | 22.6 | 7.4 |
| Bran | 2.0 | 2.0 |
| Wheat | 68.0 | 34.2 |
| Fishmeal | 1.0 | 50.0 |
| Calcium carbonate | 1.4 | 1.4 |
| Calcium bicarbonate | 1.2 | 1.2 |
| Grease | 2.0 | 2.0 |
| Premix | 1.8 | 1.8 |
| Total | 100 | 100 |
| Nutritive index |  |  |
| Metabolisable energy (MJ/kg) | 14.16 | 19.40 |
| Crude protein | 20.80 | 48.30 |
| Calcium | 1.08 | 2.68 |
| Available phosphorus | 0.48 | 1.65 |
| Lysine | 1.15 | 1.80 |
| Methionine | 0.50 | 1.15 |
| Cystine | 0.87 | 1.04 |
