# Supplementary material for: Temporal Effects of High Fishmeal Diet on Gut Microbiota and Immune Response in Clostridium perfringens-Challenged Chickens
Source: Front Microbiol. 2018 Nov 13;9:2754. doi: 10.3389/fmicb.2018.02754 (PMC6243065; doi:10.3389/fmicb.2018.02754)
Supplement: Supplementary file 2 [file Table_2.DOCX]

Table S2 Primers used for qPCR

| Target gene | Forward sequence (5’ to 3’) | Reverse sequence (5’ to 3’) | Reference |
| --- | --- | --- | --- |
| TLR2 | GGGGCTTCACTTCTCTGCTT | AGCATCCTCTGAGATTTGACG | (Monk et al., 2015) |
| NOD1 | AGCACTGTCCATCCTCTGTCC | TGAGGGTTGGTAAAGGTCTGCT | (Guo et al., 2015) |
| TNF-α | TGCTGTTCTATGACCGCC | CTTTCAGAGCATCAACGCA | (Zhao et al., 2017) |
| IL-8 | ATGAACGGCAAGCTTGGAGCTG | TCCAAGCACACCTCTCTTCCATCC |  |
| AGP | GGTGTACATCATGGGTGCCT | CGCATGTTTCATTCAGCCTCA | (Marques et al., 2017) |
| SAA | TGCTTCGTGTTGCTCTCCAT | CATGTCCCGGTATGCTCTCC |  |
| PIT54 | GCCAGTGCAATTTGTTCAGA | TCCCGTAAATCCCAGTTGTC |  |
| CRP | ATCCCATGCTCAACTTCACC | CCGACGTAGAAGCGGTACTC |  |
| OVT | AGCCATTGCGAATAATGAGG | ATGGGCTTCAGCTTGTATGG |  |
| GAPDH | TGCTGCCCAGAACATCATCC | ACGGCAGGTCAGGTCAACAA | (Zhang et al., 2017) |
| *C. perfringen*s | GGCGGTAATATATCTGTTGAAGG | ACCGTCCTTAGTCTCAAC | (Schlegel et al., 2012) |
| 16s | GCCAGCAGCCGCGGTAA | AGGGTATCTAATCCT | (Buffie et al., 2015) |

IL, interleukin; TNF-α, tumour necrosis factor α; TLR, Toll-like receptor; NOD, nucleotide-binding oligomerization domain; AGP, alpha 1- acid glycoprotein; SAA, Serum Amyloid A; CRP, C- Reactive protein; OVT, Ovotransferrin; GAPDH, glyceraldehyde-3-phosphate dehydrogenase.

**Reference**

Buffie, C.G., Bucci, V., Stein, R.R., Mckenney, P.T., Ling, L., Gobourne, A., No, D., Liu, H., Kinnebrew, M., Viale, A., Littmann, E., Van Den Brink, M.R., Jenq, R.R., Taur, Y., Sander, C., Cross, J.R., Toussaint, N.C., Xavier, J.B., and Pamer, E.G. (2015). Precision microbiome reconstitution restores bile acid mediated resistance to Clostridium difficile. *Nature* 517**,** 205-208.

Guo, S., Li, C., Liu, D., and Guo, Y. (2015). Inflammatory responses to a Clostridium perfringens type A strain and alpha-toxin in primary intestinal epithelial cells of chicken embryos. *Avian Pathol* 44**,** 81-91.

Marques, A.T., Nordio, L., Lecchi, C., Grilli, G., Giudice, C., and Ceciliani, F. (2017). Widespread extrahepatic expression of acute-phase proteins in healthy chicken (Gallus gallus) tissues. *Vet Immunol Immunopathol* 190**,** 10-17.

Monk, J.M., Zhang, C.P., Wu, W., Zarepoor, L., Lu, J.T., Liu, R., Pauls, K.P., Wood, G.A., Tsao, R., Robinson, L.E., and Power, K.A. (2015). White and dark kidney beans reduce colonic mucosal damage and inflammation in response to dextran sodium sulfate. *J Nutr Biochem* 26**,** 752-760.

Schlegel, B.J., Nowell, V.J., Parreira, V.R., Soltes, G., and Prescott, J.F. (2012). Toxin-associated and other genes in Clostridium perfringens type A isolates from bovine clostridial abomasitis (BCA) and jejunal hemorrhage syndrome (JHS). *Can J Vet Res* 76**,** 248-254.

Zhang, B., Lv, Z., Li, H., Guo, S., Liu, D., and Guo, Y. (2017). Dietary l-arginine inhibits intestinal Clostridium perfringens colonisation and attenuates intestinal mucosal injury in broiler chickens. *Br J Nutr* 118**,** 321-332.

Zhao, X., Yang, J., Wang, L., Lin, H., and Sun, S. (2017). Protection Mechanism of Clostridium butyricum against Salmonella Enteritidis Infection in Broilers. *Front Microbiol* 8**,** 1523.
